# Overview of botulinum neurotoxin-producing clostridia in soils in France

**DOI:** 10.1128/spectrum.00114-25

**Published:** 2025-06-18

**Authors:** Caroline Le Maréchal, Adeline Huneau, Sébastien Solanas, Amandine Avouac, Léa Jambou, Apolline Roux, Line Boulonne, Sandra Rouxel, Claudy Jolivet, Typhaine Poezevara, Marianne Chemaly, Alain Hartmann

**Affiliations:** 1 Ploufragan-Plouzané-Niort Laboratory, ANSES, UHQPAP, Ploufragan, France; 2 Ploufragan-Plouzané-Niort Laboratory, ANSES, UEPISABE, Ploufragan, France; 3 INRAE, UMR1347 Agroécologie 117488 , Dijon, France; 4 INRAE, UR 1508 Info&Sols 266457 , Orléans, France; Instituto de Ecología, A.C. (INECOL), Pátzcuaro, Michoacán, Mexico

**Keywords:** clostridia, *Clostridium botulinum*, botulinum neurotoxin, soil

## Abstract

Soil is a known reservoir of human pathogens, including botulinum neurotoxin-producing clostridia. However, the occurrence and distribution of botulinum neurotoxin types in French soils are unknown, prompting this study. Botulinum neurotoxin-producing clostridia were sought, and botulinum neurotoxin types were identified through an analysis of 486 soil samples representative of nationwide soil type and land use, provided by the French Soil Quality Monitoring Network. Botulinum neurotoxin-producing clostridia were detected in 45.3% of soil samples, with type B-producing clostridia being the most frequently detected (40.1% of soil samples). Geographical variability was observed, with samples from the North of France being more frequently positive than those from the South. A comparison of these detection results with the characteristics of soils and climate conditions revealed three soil clusters. *Clostridium botulinum* type A was overrepresented in the first cluster, comprising sandy soils, with a higher C:N ratio and higher yearly precipitation than in all the other soil samples. Type B-producing clostridia were overrepresented in the other two clusters, comprising silty soils in cluster 2 and clay soils in cluster 3 with a high concentration of cationic minerals and a higher cation exchange capacity and pH than the average among all samples. Finally, a non-toxic surrogate strain was seen to have survived and was monitored in different selected soils under various laboratory conditions. It remained highly stable, with persistent spores.

**IMPORTANCE:**

Botulism is a flaccid paralysis disease caused by one of the seven neuroparalytic toxins produced by anaerobic spore-forming clostridia. While soil is reported in the literature as being an important reservoir of clostridia capable of producing botulinum neurotoxins, no study has been conducted in France up to now to establish its prevalence and study soil factors influencing its occurrence. The significance of our research is in providing a global picture of the unequal distribution of botulinum neurotoxin-producing clostridia and detected subtypes countrywide, showing that types involved in human botulism outbreaks are commonly detected, while those involved in animal outbreaks are rare. Finally, the stability of spores was evaluated. Results showed a high persistence of spores under tested conditions. This study provides new data regarding the distribution of botulinum neurotoxin-producing clostridia in soils that are crucial for a better understanding and management of animal and human botulism outbreaks.

## INTRODUCTION

Botulism is a severe neuroparalytic disease due to the action of the botulinum neurotoxin (BoNT) produced by clostridia, in particular *Clostridium botulinum*. Human botulism is mostly associated with BoNTs A, B, E, and F, and animal botulism is associated with BoNTs C, D, C/D, and D/C (1). Clostridia are spore-forming bacteria, a form that enables them to resist adverse conditions and plays a key role in their survival and dispersion in the environment (2). *C. botulinum* has been reported to persist for months or even years in the environment (3–5). This ability to persist in the long term is a major issue when trying to control outbreaks of animal botulism. For instance, the cleaning and disinfection of animal buildings and equipment after a botulism outbreak is particularly challenging (6–8). The persistence of *C. botulinum* may lead to the recurrence of on-farm outbreaks (6). There have also been reports of manure being contaminated by *C. botulinum* after botulism outbreaks in poultry (9). Poultry manure is also a well known source of contamination, which can trigger botulism among cattle (8, 10–14). The management of contaminated manure is therefore particularly important due to the persistence of clostridia over time and the risk of cross-contamination between poultry and cattle. There is currently no way of eliminating *C. botulinum* in manure apart from complete incineration (7, 9). Studies are needed to assess the risks and consequences associated with the practice of spreading manure contaminated by *C. botulinum* over fields to fertilize crops, which can contaminate the soil. This could notably occur in the event of asymptomatic carriage of *C. botulinum* by animals during silent contamination.

Soil is a reservoir for various clostridia (15), including BoNT-producing clostridia (15, 16). BoNT-producing clostridia, for example, have been detected in 23.5% of soil samples from Argentina (17), 5.7% from Great Britain (18), 16.5% from Japan (19), 23.3% from Costa Rica (20), and 24.3% from the USA (21). These studies report a high variation in the detection rate of *C. botulinum* according to the regions investigated, together with variability in the BoNT type detected. While data on the distribution of BoNT-producing clostridia in soils are available for many countries (15, 17), no study had previously been conducted in France, except for an old one that evaluated the prevalence of *C. botulinum* in the Camargue wetlands (22) and another in sediments on the Canche River (23). The prevalence of BoNT-producing clostridia in French soils was thus unknown previous to our research. Besides their detection and prevalence, it is vital to evaluate the impact of biotic and abiotic parameters on the distribution and survival of BoNT-producing clostridia in soils so as to better understand their cycle in the environment. Factors such as temperature, water activity, moisture content, pH value, redox potential, oxygen concentration, biotic interactions, and nutrient availability influence the occurrence and distribution of BoNT-producing clostridia (15, 24). An improved understanding of the impact of such factors on the presence and survival of BoNT-producing clostridia will be critical to assess the risks of soil and crop contamination, depending on soil type, and thus will be able to provide recommendations on manure spreading practices.

The objective of this study was to investigate the distribution of BoNT-producing clostridia among soils in France, to study the soil and environmental parameters associated with their detection, and to assess their fate in soils (persistence and capacity to germinate) under *in vitro* conditions in the context of animal botulism.

## MATERIALS AND METHODS

### Soil sampling and soil characterization

The French Soil Quality Monitoring Network (RMQS) was set up in 2001 to monitor soil quality (soil texture, along with physical and chemical characteristics to assess soil fertility and soil contamination, and also biological parameters to assess soil biodiversity) on a representative sample of 2,200 locations based on a systematic 16 × 16 km square grid over the whole of France (25, 26). Soil samples were obtained from 486 locations monitored by the RMQS network to analyze for the presence of *C. botulinum*. Briefly, soils were collected between 2020 and 2023 following a single sampling (pool of 25 samples over a 20 × 20 m grid of the 0–30 cm soil horizon) and processed as previously described (27). Fresh soil samples were received at INRAE Genosol Platform in Dijon within a week of sampling. They were strained through a 4 mm sieve, then immediately frozen and stored below −18°C until analysis.

For 471 soils out of 486, descriptive data on physical (particle size) and chemical parameters (pH, organic C, N, exchangeable cations, and cation exchange capacity [CEC], among others) were extracted from the DONESOL database (28). These data were retrieved for each soil by INRAE’s Soil Analysis Laboratory (Arras, France). Supplementary data on climate conditions (annual average rain, annual average temperature, and evapotranspiration) were calculated from SAFRAN (Système d’Analyse Fournissant des Renseignements Adaptés à la Nivologie). Land cover for 372 soils was available in the SI SOL database by RMQS. The chemical and textural compositions of the soils, along with climate data, are shown in the supplemental material (Table S2; Fig. S1).

### Detection and enumeration of BoNT-producing clostridia

The samples were processed as previously described, with some slight modifications (29), to detect and enumerate BoNT-producing clostridia.

Briefly, 25 g of each sample soil was weighed, 10-fold diluted in pre-reduced trypticase-peptone-glucose-yeast (TPGY) extract broth (30), and homogenized using a Pulsifier blender (Microgen, Surrey, UK) for 15 s. The sample was then incubated under anaerobic conditions at 37°C for 18 h. After incubation, 1 mL of the enrichment was collected for DNA extraction using the DNeasy PowerSoil Pro kit (Qiagen) according to the manufacturer’s instructions.

Only positive samples were subject to the enumeration process using a most probable number (MPN)-PCR method. Once a 25 g sample was diluted in pre-reduced TPGY, then homogenized for 15 s using a Pulsifier blender (Microgen), the suspension was 1:5 serially diluted in 2 mL TPGY in a microplate. The serial dilution process was performed in triplicate. These dilutions were incubated at 37°C in the anaerobic chamber for 18 h. One milliliter of each dilution replicate was then collected after 18 h of incubation for DNA extraction using the DNeasy PowerSoil Pro kit (Qiagen) according to the manufacturer’s instructions.

Genes encoding BoNTs A, B, C, D, C/D, D/C, E, and F were detected using real-time PCR with a Bio-Rad CFX96 thermal cycler as previously described (29). Primers and probes are listed in Table S1.

A sample was considered positive when characteristic amplification was detected. For enumeration purposes, the MPN per gram value was estimated by an MPN calculator with a 95% confidence interval (IC_95%_).

### Bacterial strain and inoculum production

*Clostridium botulinum* group III, responsible for animal botulism, is part of the *Clostridium novyi sensu lato* genotype, along with *Clostridium novyi* and *Clostridium haemolyticum* (31), and these three are considered close relatives (32). Here, a non-toxigenic strain, called N17LNRB01, which had been isolated from the liver of a mallard duck with typical clinical signs of botulism and positive for BoNT C/D using PCR during initial diagnostic analyses, was used as a pathogen surrogate to test the survival of *C. botulinum* group III under soil conditions. *C. novyi* was grown in 1 mL of TPGY broth in each well of a 24-well microtiter plate. The plate cultures were incubated for 72 h at 37°C under anaerobic conditions (GasPak Ez, BD) in sealed jars. The culture was then washed in sterile water and concentrated (10-fold) by centrifugation to recover spores. A spore suspension containing 2 × 10^4^ spores/mL was used as inoculum for survival experiments in microcosms.

### Soil samples for survival analysis in microcosms

Four soils were chosen from the 486 RMQS soil samples (see above) based on their variable physical and chemical characteristics (i.e., different pH and clay content) (see Table 3). They were kept at 4°C until analysis (fresh soil samples).

Soil microcosms were prepared in 40 mL sterile flasks using 2 g of fresh soil adjusted to 80% of the water field capacity before inoculation (taking into account the volume of inoculum). For all tested soils, three individual microcosms were prepared for each sampling time (three biological repeats). Next, 100 µL of inoculum was added to each microcosm, giving concentrations of 1 × 10^3^ spores of N17LNRB01 per gram of dry soil. Soil microcosms were incubated at 20°C in various anaerobic or aerobic (microcosms were either exposed to air during all the experiment or not at all or were alternatively exposed to air and not exposed to air at regular time intervals) conditions and were sampled 1 h, 14 days, and 1, 3, and 6 months after inoculation. All the counts were expressed per gram of dry soil.

### *C. novyi sensu lato* enumeration in soil microcosms

Due to the unavailability of a selective medium for the enumeration of *C. novyi sensu lato*, it was enumerated in culture and in soil microcosms using a MPN-PCR method adapted from reference 29. Briefly, at each sampling time, three soil microcosms (2 g of soil) were analyzed. Each one was diluted in 18 mL of TYPG broth, homogenized by shaking at 150 rpm for 15 minutes (orbital shaker), then serially diluted in TYPG (10-fold dilution in 24-well plates). Three independent serial dilution series were completed per dilution. The plates were then incubated for 18 h at 37°C under anaerobic conditions. DNA was then extracted from 250 µL of each enrichment culture using the DNeasy PowerSoil Pro HTP 96 kit (Qiagen, France). *C. novyi sensu lato* was detected with PCR using the primers, probes, and conditions previously described (33). MPN numbers were calculated and computed using a McCrady table.

### Statistical analysis

The results of *C. botulinum* detection, soil data, climate data, and land cover data were described in terms of frequencies for qualitative variables and mean and distribution characteristics for quantitative variables using the dfsummary function of the summarytools package (version 1.0.1. [34]) in R (version 4.1.2, “Bird Hippie” [35]). The chi-square test was used to compare proportions. A principal component analysis (PCA) was carried out to discriminate soil samples according to their characteristics. It was based on data for soil texture, chemical composition, and climate conditions. PCA analysis results were classified in hierarchical ascendant order (HAC) in order to constitute clusters of soils with homogenous characteristics. Each cluster is characterized by statistically higher average values for the variables defining the cluster than the average values observed in the total sample population. Variables describing *C. botulinum* and BoNT detection in the soils were introduced as qualitative supplementary variables in the HAC analysis so we could differentiate soil clusters according to the frequency at which *C. botulinum* and BoNT were detected. The PCA and HAC processes were carried out with the FactomineR package (version 2.9 [36]).

Regarding the outcome of *C. novyi sensu lato* in microcosms, statistics were analyzed using the Kruskal-Wallis test (non-parametric statistics) to compare survival rates over time and in different soil types.

## RESULTS

### Detection of BoNT-producing clostridia in soils in France

**Fig 1:**
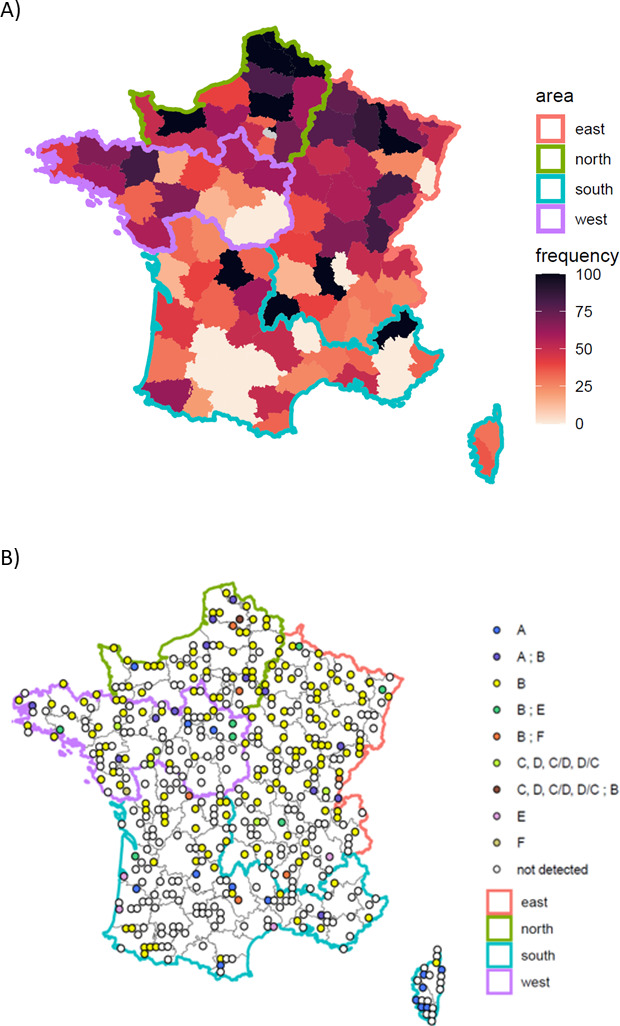
Geographical distributions of soil samples according to (A) frequency (%) of detection of BoNT-producing clostridia per department and (B) types of BoNT-producing clostridia in France (471 soil samples, 2020–2023). Maps are from GEOFLA.

BoNT-producing clostridia were detected in 220 soil samples out of 486 (45.3%, Table 1). *C. botulinum* type B was more frequently detected than the other BoNT-producing clostridia, which were detected in 195 samples (40.1%). It was associated with another BoNT-producing clostridia in 27 samples (27 out of 486, 5.5%). *C. botulinum* types C, D, C/D, or D/C were detected only in five samples (1.7%). The BoNT-producing clostridia count was lower than 13 MPN/g (IC_95%_ 4.3–39.0) in all soil samples. Detection frequencies were higher in soils collected in the North (69%) and East (53%) than in the South of France (30%) (Fig. 1).

**TABLE 1:** Detection of BoNT-producing clostridia in soils in France (*N* = 486 soil samples, 2020–2023)

Parameter	*N*	%	IC_95%_
Detection of BoNT-producing clostridia			
Yes	220	45.3	40.9-49.7
No	266	54.7	
*bont* gene detected using PCR after enrichment			
B	168	34.6	30.4-38.8
A	15	3.1	1.6-4.6
A and B	13	2.7	1.3-4.1
B and E	7	1.4	
B and F	6	1.2	
E	5	1.0	
C, D, C/D, or D/C	4	0.2	
C, D, C/D or D/C, and B	1	0.2	
F	1	0.2	
Not detected	266	54.7	

### Impact of soil characteristics on BoNT-producing clostridia

**Fig 2:**
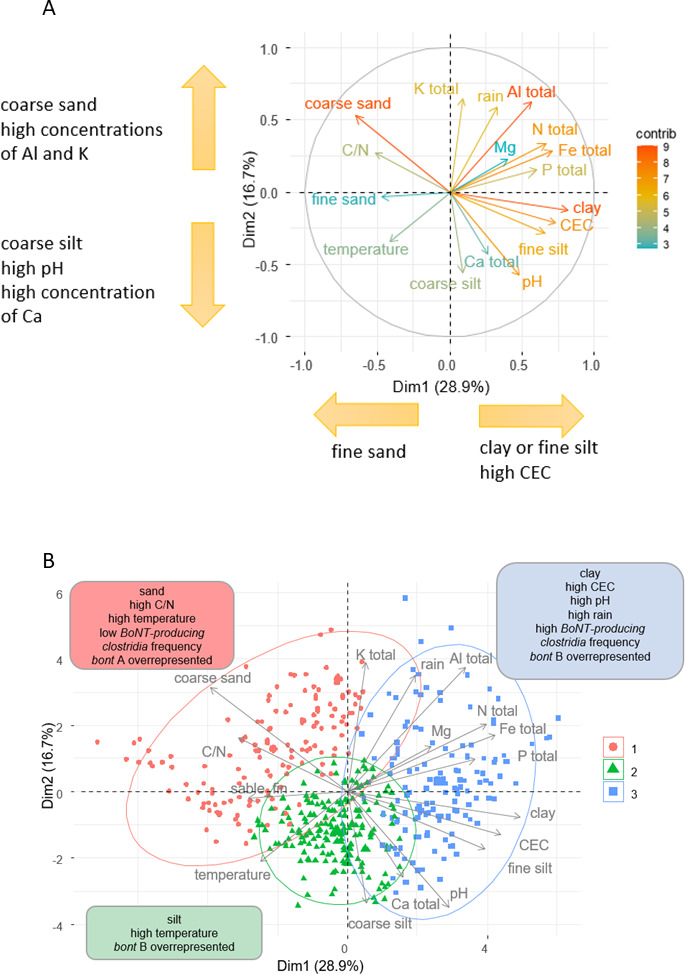
Principal component analysis (PCA) of soil characteristics and climate conditions (**A**) and characteristics of soil sample clusters obtained from hierarchical ascendant classification of principal components (**B**) (471 soil samples, France, 2020–2023). Results of the PCA are shown as contributions (contrib) of the variables to the first two dimensions of the PCA.

The main textural, chemical, and climate-related characteristics differentiating soils were identified through the PCA (Fig. 2). Based on this analysis, three soil clusters were defined using HAC (Table S3), characterized by how frequently BoNT-producing clostridia and *C. botulinum* types A and B were detected. Cluster 1 (*N* = 142) comprised sandy soils (average of sand concentration 484 g/kg versus 221 g/kg over all soils analyzed) mostly from the South of France (Corsica, Nouvelle-Aquitaine, Limousin, and Poitou-Charentes), with a higher C:N ratio (15.6 versus 12.3) and higher yearly temperatures (11.6°C versus 11.2°C) than all the other soil samples included in this study. The detection frequency for BoNT-producing clostridia in this cluster was lower (34%) than that in the total population of samples (40%), and *C. botulinum* type A was overrepresented, with a frequency of detection of 21% (versus 10% in the other soil samples). Cluster 2 (*N* = 193) comprised silty soils (average of coarse silt concentration 282 g/kg versus 182 g/kg), more frequently located in the North (Hauts-de-France and Ile-de-France) and West (Normandie, Pays-de-la-Loire, and Centre-Val-de-Loire) of France. The detection frequency in this cluster was 48%, and *C. botulinum* type B was overrepresented among the positive samples (93% versus 85% in all the positive samples). Cluster 3 (*N* = 136) comprised clay soils with a high concentration of cationic minerals (Fe, Al, Mg, and Ca), a higher CEC (25.6 versus 13.4 in the other soils), and a higher pH (7.0) than the average for all samples (6.3). The detection frequency for BoNT-producing clostridia (54%) and *C. botulinum* type B (92% of the positive samples) was also higher in this cluster than in the whole population of sampled soils. Cluster 3 comprised soil samples from the East of France (Auvergne-Rhône-Alpes, Grand-Est, Bourgogne-Franche-Comté, and Provence-Alpes-Côte-d’Azur).

### Impact of soil use on the detection of BoNT-producing clostridia

Information on land cover (Table 2) was available for 372 samples. Soils were mainly occupied by temporary crops (44%). BoNT-producing clostridia were detected in 175 samples (47%), with *C. botulinum* type B being the most frequent (158/343, 42%). BoNT-producing clostridia were detected more frequently in soils bearing temporary crops (59%) and permanent grassland (51%) than in forest soils (25%, chi-squared test, *P* < 0.01). Among temporary crops, 20 samples were from temporary grassland and ryegrass, of which 14 (70%) were positive for BoNT-producing clostridia. When applying the PCA analysis previously developed over the 372 soil samples, cluster 1 was associated with forest (40% versus 25% in the other soils) and to a lesser extent to permanent pasture (44% versus 22%), while cluster 2 was associated with temporary crops (58% versus 44%). No association was observed between cluster 3 and the other types of land cover.

**TABLE 2:** Land cover, soil texture, and detection of BoNT-producing clostridia (*N* = 343 soils, France, 2020–2023)

Land cover	*N*	%	Soil texture (g/kg) mean (SD^*a*^)			Detection of BoNT-producing clostridia (%)	Most frequent *bont* gene detected (%)^*b*^	
			Clay (<2 µm)	Silt (2–50 µm)	Sand (50–2,000 µm)		A	B
Temporary crop (cereals, oilseed, etc.)	166	45	248 (122)	498 (171)	254 (195)	98 (59)	11 (7)	92 (55)
Including temporary grassland	15	4	234 (152)	370 (169)	394 (244)	10 (66)	1 (7)	10 (67)
Permanent pasture	83	22	246 (134)	348 (147)	406 (227)	42 (51)	3 (4)	38 (46)
Forest	95	25	225 (144)	348 (180)	426 (263)	24 (25)	4 (4)	21 (22)
Orchard, vineyard, permanent crop	11	3	255 (88)	344 (98)	401 (169)	4 (36)	2 (18)	3 (27)
Wild land, garden	17	5	211 (167)	288 (156)	502 (275)	7 (41)	2 (12)	4 (24)
Total	372	100	240 (131)	412 (183)	348 (239)	175 (47)	22 (6)	158 (42)

^*a*^ Standard deviation.

^*b*^ Two *bont* genes can be detected in the same sample.

### Persistence of *C. novyi* spores over a 6-month period in four soils selected for their contrasting characteristics

**Fig 3:**
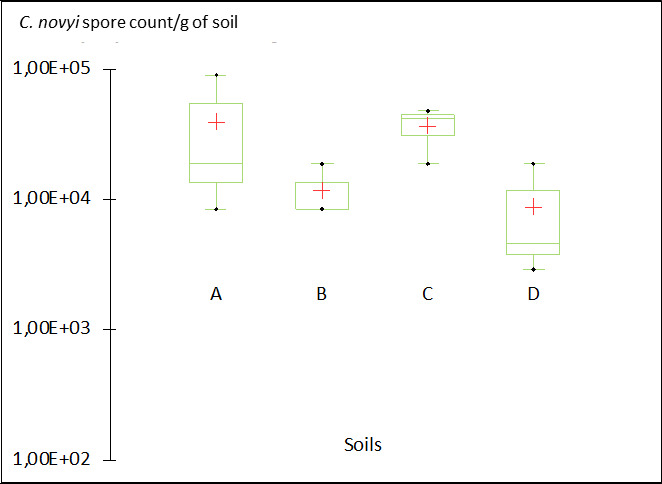
Survival of *C. novyi* in microcosms of four contrasting soils—A, B, C, and D—after 6 months of incubation under aerobic conditions at 20°C. A bilateral Kruskal-Wallis test was used to compare the distributions of spore numbers, and a bilateral Dunn test was used to estimate *P* values.

*C. novyi* was used here as a surrogate of *C. botulinum* types C, D, C/D, and D/C, all of which are involved in animal botulism outbreaks. All four soils (A, B, C, and D, as depicted in Table 3) supported the persistence of from 8.8 × 10^3^ up to 4.0 × 10^4^ spores/g of soil after 6 months’ incubation in aerobic conditions. No significant difference was observed between the number of *C. novyi* spores in the various soils (see Fig. 3) after 6 months. Persistence and capacity to germinate did not appear to differ in the four soils tested despite contrasting physical and chemical characteristics (particularly texture and pH).

**TABLE 3:** Physical and chemical characteristics of the four soils used to test the persistence of *C. novyi* spores in microcosms (Fig. 3)

Soil	Soil horizon (cm)	Clay (%)	Silt (%)	Sand (%)	pH in water	CEC	Calcium carbonate (g/kg)	C (g/kg)	N (g/kg)	Organic matter (cmol+/kg)	Soil type
A	0–25	28	39.2	32.8	8.1	13.3	656	11.7	1.87	20.2	Calcareous loam
B	0–30	9.1	28	62.9	4.5	3.87	1	8.8	0.48	ND	Sandy loam
C	0–30	16.1	53.8	30.1	6.1	6.74	0.5	13.8	1.34	23.9	Silt loam
D	0–30	23.6	29.7	46.7	7.5	16.8	19.6	25.9	2.79	44.8	Loam

**Fig 4:**
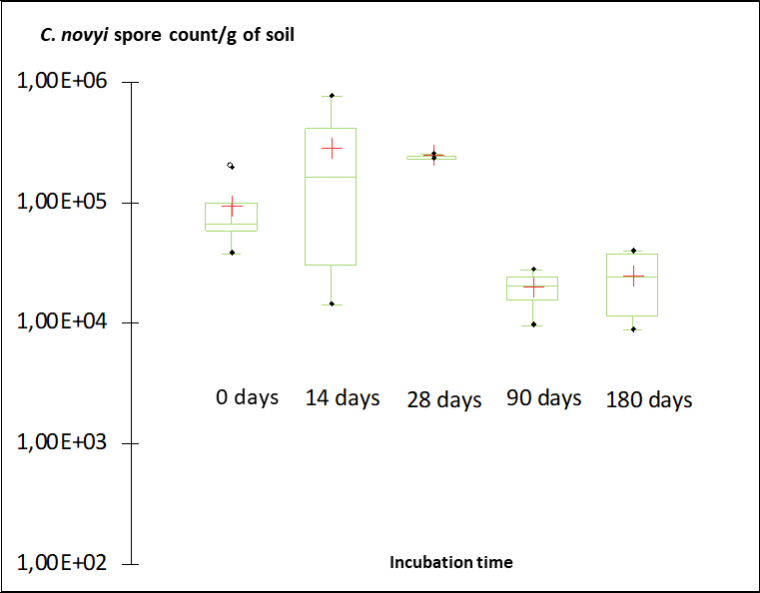
*C. novyi* spore counts in soil microcosms according to incubation time. Counts were pooled for soils A, B, C, and D at each incubation period, as depicted in Table 3. Soil microcosms were incubated at 20°C under aerobic conditions. A bilateral Kruskal-Wallis test was used to compare the numbers of spores along time, and a bilateral Dunn test was used to estimate *P* values.

The comparison of *C. novyi* spore enumeration results over the incubation period did not reveal temporal variations in the prevalence of *C. novyi* (Fig. 4) in the four soils.

### Effect of incubation conditions and soil amendment on *C. novyi* spore numbers in each soil

Neither incubation conditions (i.e., constant aerobiosis and alternating aerobiosis and anaerobiosis) nor the addition of organic matter significantly affected the persistence of *C. novyi* spores in soils. *C. novyi* spore counts were analyzed over a 6-month incubation period. After incubation of soil microcosms for a period of 6 months, no significant variation of *C. novyi* counts along time was observed in any incubation condition tested (data not shown).

## DISCUSSION

BoNT-producing clostridia were detected in 45.3% of the samples analyzed in this study. This detection level is higher than reported in previous studies conducted in other countries: 23.5% in Argentina (17), 5.7% in the United Kingdom (18), 16.5% in Japan (19), 23.3% in Costa Rica (20), and 24.3% in the USA (21). A recent review showed that *C. botulinum* spores were recovered from 19.7% of soil samples worldwide (16). The methods used to detect BoNT-producing clostridia vary among studies, which may play a role in the variability observed in the detection rate. There is no international standard on the detection of BoNT-producing clostridia in environmental samples, so each study proposes its own protocol. While the absence of a standardized method may play a role in the detection rate, the marked heterogeneity in the detection of BoNT-producing clostridia observed between regions in the present study suggests that other parameters, such as soil characteristics or climate conditions (such as temperature or precipitation), may also play a role in inter-country variability. The inter-regional differences we found echo the study conducted in Argentina, where the distribution of *C. botulinum* was not homogenous throughout the country (17). A higher level of detection was observed in the North of France (69%) than in the South (30%). Moreover, we found a higher detection rate of BoNT-producing clostridia in field crops or grassland than in forest soils. Other studies have reported similar results. The prevalence of detection in cultivated fields was 41% in a study carried out in Denmark, while no positive samples were detected in virgin soils (37). In the study carried out in Argentina, *C. botulinum* was detected more frequently in cultivated, urbanized, or industrialized soils than in soils in their natural state, unchanged by human activities (17).

*C. botulinum* type B was the most frequently detected BoNT-producing clostridia (in 40.1% of the soils analyzed in this study). It was also detected in farmed and forested soils in Denmark (37) and in the principal cattle and agricultural area of Argentina (17). In our study, *C. botulinum* type A was detected particularly in Corsica (6 out of 24 samples with *C. botulinum* type A detected, 25%). It was also detected in Italy and in 56.7% of soils in Argentina (17, 38). In the literature, data show that mainland soils are associated with type A (43%) and B (32%) worldwide (16).

*C. botulinum* types C, D, C/D, and D/C were only detected in five samples from five different *départements* (a French administrative area) in our study, with no direct link with livestock production or soil characteristics. Likewise, the study conducted in Argentina did not detect any *C. botulinum* of type C, D, C/D, or D/C (17). On the contrary, *C. botulinum* type C was detected in 11.3% of soil samples in Japan (19). In the same study, no sample collected in China revealed the presence of *C. botulinum* type C or D, showing a disparity among areas (19). *C. botulinum* type C was found only in the acidic soil of the Gulf Coast in the USA, and *C. botulinum* type D was found in alkaline soil of some western states (39). *C. botulinum* type C or D was detected in 5 soil samples out of 60 in a former cattle market in the United Kingdom (40). *C. botulinum* type C has already been detected in soils or sediments in Canadian wetlands, in areas with a history of botulism (4), as well as in soils from tropical countries such as Bangladesh (37), Brazil (41), and Indonesia (42).

While BoNT-producing clostridia were detected in slightly less than half of our samples, enumeration revealed a small quantity of bacteria (less than 13 MPN/g). These results are in accordance with a previous study that reported 25 cells/g for *C. botulinum* type A and 10 cells/g for *C. botulinum* type B in soil samples from China (19).

Based on the physical and chemical characteristics of soils and climate conditions, three clusters were identified in our study (Fig. 2). *C. botulinum* was more frequently found in clay soils with a high cation exchange capacity, a higher pH than in other soils, and high precipitation. *C. botulinum* type B was found mostly in silty clay soils, while *C. botulinum* type A appears to be associated with sandy soils and high average temperatures. Among these three clusters (Fig. 2B), the “higher temperature” parameter was associated with the presence of *C. botulinum* types A and B. Warmer temperatures may, in fact, be conducive to higher metabolic activity in the soil and therefore an increase in oxygen demand, which could lead to anoxic conditions, particularly in wet soils. The optimal temperatures for the growth of BoNT-producing clostridia varied between physiologically distinct groups of *C. botulinum* (I versus II), which could explain why toxigenic types vary according to a region’s climate conditions (15). Soil type and structure impact bacterial prevalence, survival, and movement in all soils (15). To the best of our knowledge, no previous study has ever investigated the link between soil structure and the presence of BoNT-producing clostridia.

Regarding pH, it has been reported that botulism outbreaks in wildlife are associated with water pH between 7.5 and 9.0 (43). Regarding soil pH, contrasting results have been published. A first study showed a link between higher amounts of *C. botulinum* types A and B and a neutral to alkaline pH (44). A second study showed a higher prevalence of *C. botulinum* type A in neutral to alkaline soils (average pH 7.5) and type B in more acidic soils (average pH 6.25) (39). Growth of *C. botulinum* types A, B, E, and F is reported to be inhibited in pH ranges below 5.0 for *C. botulinum* group II and 4.3 for *C. botulinum* group I (45).

Our study applied a direct approach using *C. novyi* as a pathogen surrogate of *C. botulinum* C, D, C/D, and D/C to inoculate soil microcosms incubated under laboratory conditions. We used several contrasting soils to see whether the soil’s physical and chemical parameters could affect their persistence.

The results obtained by this approach confirm that spores can withstand a great variety of environmental conditions, which is in agreement with the high prevalence (45% of soils) of BoNT-producing clostridia observed in the first part of the study. Our results are also in agreement with those obtained by Gessler and Böhnel (46), who demonstrated the persistence of *C. botulinum* type B in soil amended with contaminated compost over a 757 day period. *C. novyi* spores added to soils either accidentally or through amendments containing contaminated organic products persist at least 6 months even under aerobic conditions. This result is important for the management of recycled organic waste in agriculture when this waste is contaminated by BoNT-producing clostridia. Once introduced into the soil, BoNT-producing clostridia can persist over extended periods. Taken together, our results support the fact that biosecurity measures concerning organic waste recycling should take into account potentially long persistence of BoNT-producing clostridia spores once introduced into the soil.

Our study used a survey of metadata from RMQS soil samples, focusing on the occurrence and nature of organic amendments to soils. The complexity and diversity of these amendments did not allow us to test their impact on the prevalence of BoNT-producing clostridia in soils. Further work, including more sampling sites, is necessary to determine the impact of such amendments.

### Conclusion

A PCR-based approach to detect BoNT-producing clostridia revealed that almost half of the soil samples analyzed in this study contained them. However, BoNT types varied widely, though *C. botulinum* type B was the most frequently detected. This study also revealed a low detection rate of *C. botulinum* type C, D, C/D or D/C, suggesting that it is not very common in French soils. Moreover, lab-scale tests showed that the surrogate of *C. botulinum* types C, D, C/D, and D/C persists in soils despite using a variety of test conditions. Taken all together, these results suggest that the spreading of manure contaminated by *C. botulinum* types C, D, C/D, and D/C may contaminate soils and is therefore not advisable. Further studies are required to find appropriate ways of managing contaminated manure to prevent the dissemination of *C. botulinum* types C, D, C/D, and D/C in soils and in the environment.

## SUPPLEMENTAL MATERIAL

Supplemental material spectrum.00114-25-s0001.docx Tables S1 to S8; Fig. S1.

ASM does not own the copyrights to Supplemental Material that may be linked to, or accessed through, an article. The authors have granted ASM a non-exclusive, world-wide license to publish the Supplemental Material files. Please contact the corresponding author directly for reuse.
