# Supplementary material for: Overview of botulinum neurotoxin-producing clostridia in soils in France
Source: Microbiol Spectr. 2025 Jun 18;13(8):e00114-25. doi: 10.1128/spectrum.00114-25 (PMC12323636; doi:10.1128/spectrum.00114-25)
Supplement: Supplemental material — Tables S1 to S8; Fig. S1. [file spectrum.00114-25-s0001.docx]

Overview of botulinum neurotoxin-producing clostridia in soils in France

Supplementary data

Table S1: primers and probes used in the study for *bont* detection

| Target | | Sequence | Reference |
| --- | --- | --- | --- |
| A | Primer-Forward | GGAGTCACTTGAAGTTGATACAAATC | Fach et al. 2009 |
|  | Primer-Reverse | GCTAATGTTACTGCTGGATCTGTAG |  |
|  | Probe | Hex-TCTTTTAGGTGCAGGCAAATTT-BHQ1 |  |
| B | Primer-Forward | GATGAACAGCCAACATATAGTTGTCA |  |
|  | Primer-Reverse | GTTTCCTTTTTACCTCTTTTAAGTACCATT |  |
|  | Probe | Hex-TGATGAKATAGGATTGATTGGTATTCA-BHQ1 |  |
| E | Primer-Forward | CTATCCAAAATGATGCTTATATACCAAA |  |
|  | Primer-Reverse | GGCACTTTCTGTGCATCTAAATA |  |
|  | Probe | Hex-ATGATTCTAATGGAACAAGTGATATAGAACAACATGATGT-BHQ1 |  |
| F | Primer-Forward | GCAATATAGGATTACTAGGTTTTCATTC |  |
|  | Primer-Reverse | GAAATAAAACTCCAAAAGCATCCATT |  |
|  | Probe | Hex-TTGGTTGCTAGTAGTTGGTATTATAACAA-BHQ1 |  |
| ntnh | Primer-Forward | TACTGATTTATTTAGACCTGATTG | Woudstra et al. 2015 |
|  | Primer-Reverse | CTTTAGGCACCTTGTTATTG |  |
|  | Probe | Hex-TCATCCAATTATAATCTCCATCTCTAAGTGA-BHQ1 |  |
| Novyi-sensu-lato | Primer-Forward | GGAACCAACCTACCGAG |  |
|  | Primer-Reverse | GTAGCCACCTCCTTAACA |  |
|  | Probe | Hex-TCCAGACACGAAGAGGCTTAATATATCCA-BHQ1 |  |

Supplementary material 2: Soil characteristics and climatic conditions

Table S2: textural and chemical characteristics of soils of the RMQS network analysed for C. botulinum detection and climatic conditions associated to the soil locations (France, 2020-2023)

|  | parameter | mean | sd | min | med | max | method | unit | detection threshold |
| --- | --- | --- | --- | --- | --- | --- | --- | --- | --- |
|  | clay (< 2µm) | 236 | 132 | 12 | 200 | 707 | NF X 31-107 | g/kg |  |
| Texture | silt (2-20 µm) | 226 | 98 | 1 | 234 | 482 | NF X 31-107 | g/kg |  |
|  | silt (20-50 µm) | 182 | 117 | 1 | 151 | 518 | NF X 31-107 | g/kg |  |
|  | sand (50-200 µm) | 134 | 8 | 8 | 113 | 677 | NF X 31-107 | g/kg |  |
|  | sand (200 - 2000 µm) | 221 | 215 | 2 | 138 | 927 | NF X 31-107 | g/kg |  |
| Chemical composition | cation exchange capacity | 13,4 | 11,2 | 0,2 | 9,1 | 70,1 | NF X 31-130 | cmol+/kg | 1 |
|  | exchangeable calcium | 11,8 | 11,6 | 0 | 7,5 | 60,5 | NF X 31-130 | cmol+/kg | 0,01 |
|  | exchangeable potassium | 0,4 | 0,3 | 0 | 0,3 | 2,1 | NF X 31-130 | cmol+/kg | 0,02 |
|  | exchangeable magnesium | 1,1 | 1,8 | 0 | 0,7 | 20,2 | NF X 31-130 | cmol+/kg | 0,005 |
|  | exchangeable sodium | 0,1 | 0,4 | 0 | 0 | 6,8 | NF X 31-130 | cmol+/kg | 0,005 |
|  | exchangeable manganese | 0,1 | 0,1 | 0 | 0 | 2,1 | NF X 31-130 | cmol+/kg | 0,005 |
|  | exchangeable aluminium | 0,6 | 1,2 | 0 | 0,1 | 8,8 | NF X 31-130 | cmol+/kg | 0,02 |
|  | exchangeable iron | 0 | 0 | 0 | 0 | 0,1 | NF X 31-130 | cmol+/kg | 0,005 |
|  | available phosphorus | 0,1 | 0,1 | 0 | 0 | 0,6 | Olsen method | g/kg | 0,005 |
|  | total limestone | 47,3 | 125,1 | 0,5 | 0,5 | 739 | NF ISO 10693 | g/kg | 1 |
|  | pH | 6,3 | 1,3 | 3,8 | 6,2 | 8,6 | NF ISO 10390 |  |  |
|  | organic carbon | 24,4 | 20,2 | 4,5 | 19 | 243 | dry combustion | g/kg | 0,05 |
|  | total nitrogen | 2 | 1,5 | 0,2 | 1,7 | 16 | dry combustion | g/kg | 0,02 |
|  | organic matter | 44,3 | 36,7 | 7,8 | 34,3 | 420 | dry combustion | g/kg | 0,086 |
|  | available iron | 0,4 | 0,2 | 0 | 0,3 | 1,9 | Tamm method | g/100g | 0,008 |
|  | total aluminium | 4,8 | 2,1 | 0,1 | 4,7 | 10 | NF X 31-147 | g/100g | 0,02 |
|  | total calcium | 2,3 | 5 | 0 | 0,4 | 29,3 | NF X 31-147 | g/100g | 0,02 |
|  | total iron | 2,5 | 1,5 | 0 | 2,3 | 9,2 | NF X 31-148 | g/100g | 0,02 |
|  | total magnesium | 0,5 | 0,9 | 0 | 0,3 | 10,5 | NF X 31-149 | g/100g | 0,02 |
|  | total manganese | 676 | 523 | 5 | 575 | 4930 | NF X 31-150 | mg/kg | 10 |
|  | total potassium | 1,6 | 0,9 | 0 | 1,4 | 4,4 | NF X 31-151 | g/100g | 0,02 |
|  | total sodium | 0,6 | 0,5 | 0 | 0,4 | 3 | NF X 31-152 | g/100g | 0,02 |
|  | phosphates | 0,2 | 0,1 | 0 | 0,2 | 1,1 | NF X 31-153 | g/100g | 0,001 |
|  | soluble boron | 0,2 | 0,2 | 0 | 0,2 | 0,4 | NF X 31-122 | mg/kg | 0,05 |
|  | total cadnium | 0,3 | 0,3 | 0 | 0,2 | 3,1 | NF X 31-147 | mg/kg | 0,02 |
|  | extractable cadnium | 0,1 | 0,2 | 0 | 0,1 | 1,4 | EDTA, method BCR | mg/kg | 0,01 |
|  | total cobalt | 10,9 | 9,5 | 0,5 | 9,3 | 100 | NF X 31-147 | mg/kg | 1 |
|  | total chromium | 62 | 157 | 2 | 47 | 3030 | NF X 31-147 | mg/kg | 1 |
|  | extractable chronium | 0,2 | 0,2 | 0 | 0,1 | 3,5 | EDTA, method BCR | mg/kg | 0,02 |
|  | total copper | 18 | 17 | 0,5 | 14 | 178 | NF X 31-147 | mg/kg | 1 |
|  | extractable copper | 4 | 8,4 | 0,1 | 2,3 | 78,4 | EDTA, method BCR | mg/kg | 0,05 |
|  | total molybdenum | 0,8 | 1,1 | 0 | 0,6 | 19,8 | NF X 31-147 | mg/kg | 0,04 |
|  | total nickel | 29 | 86 | 0,5 | 18 | 1530 | NF X 31-147 | mg/kg | 1 |
|  | extractable nickel | 1,4 | 4,4 | 0 | 0,7 | 76,3 | EDTA, method BCR | mg/kg | 0,1 |
|  | total lead | 33,1 | 33,3 | 3,2 | 28,1 | 624 | NF X 31-147 | mg/kg | 0,1 |
|  | extractable lead | 7,4 | 8,9 | 0,6 | 5,6 | 135 | EDTA, method BCR | mg/kg | 0,2 |
|  | total thallium | 0,7 | 0,9 | 0 | 0,5 | 16,8 | NF X 31-147 | mg/kg | 0,01 |
|  | total zinc | 73 | 54 | 2,5 | 61 | 532 | NF X 31-147 | mg/kg | 3 |
|  | extractable zinc | 3 | 3,9 | 0,2 | 2 | 54,8 | EDTA, method BCR | mg/kg | 0,1 |
|  | total arsenic | 16,6 | 18,6 | 0,6 | 11,9 | 249 | NF X 31-147 | mg/kg | 0,1 |
|  | total mercury | 0,1 | 0,1 | 0 | 0 | 0,8 | dry combustion | mg/kg | 0,0025 |
|  | ratio C/N | 12,3 | 4,8 | 5,1 | 10,6 | 52,7 |  |  |  |
| Climatic conditions | average rain - year | 923 | 255 | 561 | 851 | 2085 |  | mm |  |
|  | average temperature - year | 11,2 | 2 | 1,3 | 11,1 | 16,4 |  | °C |  |
|  | number days over 5°C | 295 | 35 | 113 | 299 | 362 |  |  |  |
|  | potential evapotranspiration - year | 814 | 121 | 613 | 782 | 1309 |  | mm |  |

Supplementary data : Figure S1


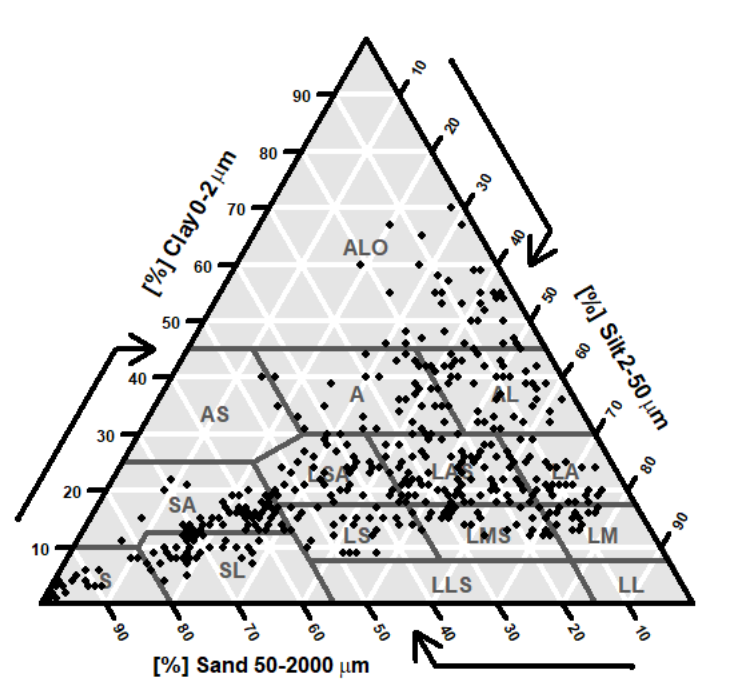


Figure S1 : Textural classification of the 471 soils (France, 2020-2023) – classification according INRAE 1995 classification (Baize and Jabiol, 1995). Created with the ‘Soil texture wizard’ package in R (Moeys, 2023)

**References**

Moeys J., 2023. The soil texture wizard: R functions for plotting, classifying, transforming and exploring soil texture data.

<https://cran.r-project.org/web/packages/soiltexture/vignettes/soiltexture_vignette.pdf>

Baize D. and Jabiol B. 1995. Guide pour la description des sols. col. Techniques et pratiques. INRA, 1995. 375 p.

Supplementary material 3: results of the PCA and HAC analysis on soil sample characteristics

**Table S3: results for Quantitative variables for Cluster 1 (142 soil samples)**

| Parameter | Cluster | | Global | | unit | P |
| --- | --- | --- | --- | --- | --- | --- |
|  | mean | sd | mean | sd |  |  |
| sand (200 - 2000 µm) | 484 | 188 | 221 | 25 | g/kg | <0.001 |
| ratio C/N | 15.6 | 7.0 | 12.3 | 4.8 |  | <0.001 |
| total potassium | 2.0 | 1.2 | 1.6 | 0.8 | g/100g | <0.001 |
| sand (50-200 µm) | 178 | 113 | 134 | 93 | g/kg | <0.001 |
| average temperature | 11.6 | 1.9 | 11.2 | 1.9 | ° C | <0.001 |
| total magnesium | 0.36 | 0.37 | 0.55 | 0.89 | g/100g | 0.001 |
| total nitrogen | 1.5 | 0.96 | 2.0 | 1.5 | g/100g | <0.001 |
| total calcium | 0.58 | 1.5 | 2.3 | 5.0 | g/100g | <0.001 |
| phosphates | 0.13 | 0.09 | 0.17 | 0.11 | g/kg | <0.001 |
| total iron | 1.8 | 1.3 | 2.5 | 1.5 | g/100g | <0.001 |
| cation exchange capacity | 6.1 | 4.0 | 13.4 | 11.2 | g/100g | <0.001 |
| pH | 5.4 | 1.0 | 6.3 | 1.3 |  | <0.001 |
| clay (< 2µm) | 130 | 65 | 236 | 132 | g/kg | <0.001 |
| silt (20-50 µm) | 86 | 51 | 182 | 117 | g/kg | <0.001 |
| silt (2-20 µm) | 122 | 63 | 226 | 98 | g/kg | <0.001 |

**Table S4: results for Qualitative modalities for Cluster 1 (142 soil samples)**

| parameter | % in cluster | % global | P |
| --- | --- | --- | --- |
| Corsica | 13 | 4 | <0.001 |
| *bont* gene B non-detected | 75 | 60 | <0.001 |
| BoNT-producing clostridia non-detected | 66 | 54 | <0.001 |
| Aquitaine-Limousin-Poitou-Charentes | 23 | 15 | <0.001 |
| *bont* gene A detected | 6 | 3 | <0.001 |
| Normandie | 2 | 5 | 0.04 |
| Nord-Pas-de-Calais-Picardie | 1 | 5 | 0.02 |
| Alsace-Champagne-Ardennes-Lorraine | 6 | 11 | 0.01 |

**Table S5: results for Quantitative variables for Cluster 2 (193 soil samples)**

| Parameter | Cluster | | Global | | unit | P |
| --- | --- | --- | --- | --- | --- | --- |
|  | mean | sd | mean | sd |  |  |
| silt (2-20 µm) | 282 | 106 | 182 | 117 | g/kg | <0.001 |
| silt (20-50 µm) | 265 | 69 | 226 | 98 | g/kg | <0.001 |
| average temperature | 11.5 | 1.1 | 11.2 | 1.9 | ° C | 0.001 |
| phosphates | 0.15 | 0.06 | 0.17 | 0.11 | g/kg | <0.001 |
| clay (< 2µm) | 210 | 59 | 236 | 132 | g/kg | <0.001 |
| total magnesium | 0.31 | 0.17 | 0.55 | 0.89 | g/100g | <0.001 |
| total iron | 2.1 | 0.80 | 2.5 | 1.5 | g/100g | <0.001 |
| cation exchange capacity | 10.2 | 5.0 | 13.4 | 11.2 | g/100g | <0.001 |
| ratio C/N | 10.7 | 2.3 | 12.3 | 4.8 |  | <0.001 |
| total potassium | 1.3 | 0.4 | 1.6 | 0.85 | g/100g | <0.001 |
| total precipitation | 829 | 145 | 923 | 254 | mm | <0.001 |
| total nitrogen | 1.5 | 0.6 | 2.0 | 1.5 | g/100g | <0.001 |
| total aluminium | 4.0 | 1.1 | 4.8 | 2.1 | g/100g | <0.001 |
| sand (200 - 2000 µm) | 108 | 85 | 221 | 215 | g/kg | <0.001 |

**Table S6: results for Qualitative modalities for Cluster 2 (193 soil samples)**

| parameter | % in cluster | % global | P |
| --- | --- | --- | --- |
| Nord-Pas-de-Calais-Picardie | 10 | 5 | <0.001 |
| Normandie | 10 | 5 | <0.001 |
| Pays de la Loire | 10 | 7 | <0.001 |
| Ile de France | 6 | 3 | <0.001 |
| Centre Val de Loire | 10 | 7 | <0.001 |
| *bont* gene B detected | 40 | 35 | 0.01 |
| *bont* gene A detected | 1 | 3 | 0.01 |
| Aquitaine-Limousin-Poitou-Charentes | 11 | 15 | <0.001 |
| Corsica | 0 | 4 | <0.001 |
| Auvergne-Rhône-Alpes | 4 | 11 | <0.001 |

**Table S7: results for Quantitative variables for Cluster 3 (136 soil samples)**

| Parameter | Cluster | | Global | | unit | P |
| --- | --- | --- | --- | --- | --- | --- |
|  | mean | sd | mean | sd |  |  |
| clay (< 2µm) | 385 | 125 | 236 | 132 | g/kg | <0.001 |
| cation exchange capacity | 25.6 | 12.8 | 13.4 | 11.2 | g/100g | <0.001 |
| total iron | 3.9 | 1.5 | 2.5 | 1.5 | g/100g | <0.001 |
| total nitrogen | 3.4 | 1.9 | 2.0 | 1.5 | g/100g | <0.001 |
| phosphates | 0.25 | 0.15 | 0.17 | 0.11 | g/kg | <0.001 |
| total aluminium | 6.1 | 1.7 | 4.8 | 2.1 | g/100g | <0.001 |
| total magnesium | 1.1 | 1.5 | 0.55 | 0.89 | g/100g | <0.001 |
| pH | 7.0 | 1.1 | 6.3 | 1.3 |  | <0.001 |
| silt (2-20 µm) | 281 | 76 | 226 | 98 | g/kg | <0.001 |
| total precipitation | 1059 | 334 | 923 | 254 | mm | <0.001 |
| ratio C/N | 4.3 | 5.8 | 2.3 | 5.0 |  | <0.001 |
| total calcium | 11.1 | 2.4 | 12.3 | 4.8 | g/100g | <0.001 |
| silt (20-50 µm) | 139 | 55 | 182 | 117 | g/kg | <0.001 |
| average temperature | 10.2 | 2.5 | 11.2 | 1.9 | ° C | <0.001 |
| sand (50-200 µm) | 87 | 58 | 134 | 93 | g/kg | <0.001 |
| sand (200 - 2000 µm) | 107 | 99 | 221 | 215 | g/kg | <0.001 |

**Table S8: results for Qualitative modalities for Cluster 3 (136 soil samples)**

| parameter | % in cluster | % global | P |
| --- | --- | --- | --- |
| Auvergne-Rhône-Alpes | 19 | 11 | <0.001 |
| Alsace-Champagne-Ardennes-Lorraine | 18 | 11 | <0.001 |
| *bont* gene B detected | 49 | 40 | 0.02 |
| BoNT-producing clostridia detected | 54 | 45 | 0.03 |
| Provence-Alpes-Côte d’Azur | 7 | 4 | 0.04 |
| Bourgogne-Franche-Comté | 16 | 11 | 0.04 |
| Ile de France | 1 | 4 | 0.03 |
| Normandie | 1 | 5 | 0.02 |
| Pays de la Loire | 2 | 7 | <0.001 |
| Nord-Pas-de-Calais-Picardie | 1 | 5 | <0.001 |
| Centre Val de Loire | 2 | 7 | <0.001 |
